# Constitutive expression of transcription factor SirZ blocks pathogenicity in *Leptosphaeria maculans* independently of sirodesmin production

**DOI:** 10.1371/journal.pone.0252333

**Published:** 2021-06-10

**Authors:** Andrew S. Urquhart, Candace E. Elliott, Wei Zeng, Alexander Idnurm

**Affiliations:** 1 School of BioSciences, The University of Melbourne, Melbourne, Victoria, Australia; 2 Applied BioSciences, Macquarie University, Macquarie Park, New South Wales, Australia; 3 Biosecurity Operations Division, Department of Agriculture, Water and the Environment, Post Entry Quarantine, Mickleham, Victoria, Australia; 4 Sino-Australia Plant Cell Wall Research Centre, State Key Laboratory of Subtropical Silviculture, Zhejiang A&F University, Hangzhou, China; Ruhr-Universitat Bochum, GERMANY

## Abstract

Sirodesmin, the major secondary metabolite produced by the plant pathogenic fungus *Leptosphaeria maculans* in vitro, has been linked to disease on *Brassica* species since the 1970s, and yet its role has remained ambiguous. Re-examination of gene expression data revealed that all previously described genes and two newly identified genes within the *sir* gene cluster in the genome are down-regulated during the crucial early establishment stages of blackleg disease on *Brassica napus*. To test if this is a strategy employed by the fungus to avoid damage to and then detection by the host plant during the *L*. *maculans* asymptomatic biotrophic phase, sirodesmin was produced constitutively by overexpressing the *sirZ* gene encoding the transcription factor that coordinates the regulation of the other genes in the *sir* cluster. The *sirZ* over-expression strains had a major reduction in pathogenicity. Mutation of the over-expression construct restored pathogenicity. However, mutation of two genes, *sirP* and *sirG*, required for specific steps in the sirodesmin biosynthesis pathway, in the *sirZ* over-expression background resulted in strains that were unable to synthesize sirodesmin, yet were still non-pathogenic. Elucidating the basis for this pathogenicity defect or finding ways to overexpress *sirZ* during disease may provide new strategies for the control of blackleg disease.

## Introduction

*Leptosphaeria maculans* is the most significant biological threat to the global canola industry [1]. This fungal pathogen displays a complex hemibiotrophic life cycle, which begins with an initial biotrophic infection of cotyledons or young leaves followed by lesion formation. The fungus travels via the petiole into the stem, and down the plant to the base of the stem where it causes cankering, and hence the common name of the disease blackleg [2].

**Fig 1:**
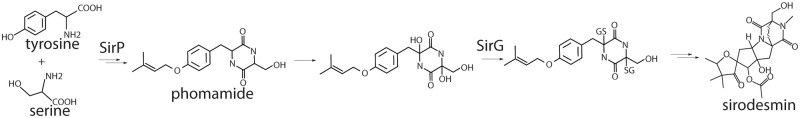
Abbreviated pathway for sirodesmin synthesis to illustrate where the enzymes SirG and SirP are predicted to function. Double arrows indicate multiple enzymatic reactions.

Like many filamentous ascomycetes involved in plant disease [3], *L*. *maculans* produces secondary metabolites of which the best studied is a related set of epipolythiodioxopiperazines, the sirodesmins. Sirodesmin PL is the prominent product, and here for simplicity (unless stated) this has been abbreviated to sirodesmin. Sirodesmin is produced by the actions of proteins encoded by a cluster of 18 reported *sir* genes [4]. Disruption of one of these genes, encoding the two-module non-ribosomal peptide synthase SirP, blocks sirodesmin biosynthesis [4]. While it was initially hypothesized that SirP catalyzed the first step in the pathway to join together tyrosine and serine [4], other experiments suggest that SirD first produces 4-*O*-dimethylallyl-l-tyrosine to which SirP then adds serine [5]. In either scenario, SirP acts in the early steps of the pathway (Fig 1).

The role of a second enzyme, SirG, in sirodesmin synthesis has not been experimentally determined. Gardiner *et al*. 2004 proposed a role in self-protection; however, based on the function of the homologous *gliG* gene, found in the gene cluster for the biosynthesis of the epipolythiodioxopiperazine gliotoxin in *Aspergillus fumigatus* [6], SirG is hypothesized to act after the synthesis of the precursor phomamide [7] (see Fig 1). The third gene studied here encodes the putative transcription factor SirZ, which is required for regulating the expression of the genes in the *sir* cluster [8, 9].

The finding that purified sirodesmin causes chlorotic lesions on canola leaves led to the hypothesis that sirodesmin was important for blackleg disease progression [10]. However, isolates in which sirodesmin production is disrupted are still able to cause normal disease on cotyledons, suggesting that these phytotoxic properties of sirodesmin are not required for early disease symptoms [4, 8, 11]. The fact that sirodesmin is indeed produced by the fungus and is stable in *B*. *napus* raised the possibility that perhaps sirodesmin was required for causing stem cankering; however, inoculation of *B*. *napus* with strains carrying the *sirP* gene mutation resulted in mildly reduced stem canker symptoms with an associated 50% reduction in fungal biomass [12]. Sirodesmin triggers the synthesis of antifungal phytoalexins when applied to *Brassica* species [13], including brassilexin, which is a plant defense compound known to be active against fungi. Hence, how sirodesmin may be involved in the pathogenicity of *L*. *maculans* is unclear.

Transcriptomic studies have found that the expression levels of a large number of *L*. *maculans* genes change at different stages of the disease cycle, including genes that are markedly down-regulated during the early stages when the fungus grows as a biotroph [14–17]. Here, we discover that the genes comprising the *sir* gene cluster responsible for the production of sirodesmin are repressed to a very low level during the early stages of disease relative to *in vitro*. We thus formulated the hypothesis that the down-regulation of this gene cluster *in planta* may be required to avoid detection by the plant. To address this hypothesis, we constitutively expressed the SirZ transcription factor and analyzed this strain and those derived from it for their ability to cause blackleg disease.

## Materials and methods

### Growth of *L*. *maculans* strains, pathogenicity and competition testing

*L*. *maculans* strains were cultured in 10% cleared V8 juice (Campbell’s) that had been adjusted to pH 6 using NaOH prior to autoclaving (CV8). 2% agar was added to produce solid media. Cultures on solid media were kept at 22°C with a 12 h light-dark cycle to induce sporulation while liquid cultures were grown at 22°C in the dark. The wild type strain used was D5. This strain was isolated in Penshurst, Victoria, in 1988 and is part of the International Blackleg of Crucifers Network collection (strain IBCN18, also called M1).

The ability of strains to cause disease was conducted on canola *Brassica napus* cv. Westar or *B*. *juncea* cv. Aurea, with lesion areas measured 14 days post inoculation, as described previously [18]. These are two *Brassica* cultivars routinely used for assessing blackleg symptoms; the seed used were obtained from previous bulking production in the laboratory. Antimicrobial activity in the strains was tested against bacterium *Bacillus subtilis* and fungus *Leptosphaeria biglobosas* as described in detail previously [12].

### RNA-sequencing expression analysis

Previously generated RNA-sequencing data were used to examine the expression of genes in the sirodesmin biosynthesis cluster. As in Urquhart and Idnurm [19], raw RNA-sequencing reads from two studies compareing *L*. *maculans* transcript levels *in vitro* and *in planta* [14, 15] were obtained from the sequence read archive at NCBI, and mapped to the *L*. *maculans* strain JN3 genome sequence [20] using Geneious version 11 software. The TPM (Transcripts Per Kilobase Million) values were calculated for the 3´ exon of each gene to minimize bias effects of long transcripts.

### Generation of strains to over-express *sirZ* during plant disease

The recombinant strains created in this study, starting in the wild type strain D5, are listed in Table 1. Oligonucleotide primers used to make constructs for transformation were synthesized by Sigma-Aldrich, Australia, with the sequence details provided in S1 Table. A construct to constitutively express SirZ was developed by cloning the coding region of the *sirZ* gene, amplified using primers AU226 and AU227, into the *Bgl*II site of plasmid PLAU2 [21] to produce plasmid PLAU50. This resulted in the *sirZ* gene being expressed under the control of the *L*. *maculans* actin promoter. Similarly, genes encoding green fluorescent protein (GFP) and the avirulence protein AvrLm6 were amplified using primer pairs AU24 and AU27; and AU28 and AU31, respectively, and cloned into the *Bgl*II site of PLAU2 for the transformation of *L*. *maculans* to create control strains. Cloning used Gibson assembly and subsequent transformation into *Escherichia coli* strain NEB^®^ 5-alpha (New England Biolabs, USA).

**Table 1:** Genetically modified strains of *Leptosphaeria maculans* developed in this study.

Strain name	Construct introduced	Strain background
D5+sirZOE#2-6A/B (i.e. 5 independent transformants derived from two separate transformation experiments, A and B).	PLAU50, for SirZ overexpression	D5 (wild type)
D5+AvrLm6	PLAU14, for AvrLm6 overexpression	D5 (wild type)
D5+GFP	PLAU17, for GFP expression as a control	D5 (wild type)
D5+sirZOE#6+cas9#2	pMAI23, expresses Cas9	D5+sirZOE#6
sirZ CRISPR	sirZ gRNA	D5+sirZOE#6+cas9#2
sirP CRISPR	sirP gRNA	D5+sirZOE#6+cas9#2
sirG CRISPR	sirG gRNA	D5+sirZOE#6+cas9#2

The constructs were transformed by electroporation into *Agrobacterium tumefaciens* strain EHA105 as described previously [18]. Strain EHA105 is a commonly used host for subsequent transformation of plants and fungi [22]. Fungal transformation was conducted on *Agrobacterium* induction medium, followed by an overlay with 200 μg/ml of hygromycin and 200 μg/μl of cefotaxime in CV8 medium [23]. Multiple independent transformants were isolated onto selection medium, and then assessed for their pathogenicity on canola.

### Generation of gene disruption strains by CRISPR-Cas9 mutations

One of the SirZ over-expression strains (6B) was selected for the construction of additional mutations. To enable genes in the sirodesmin biosynthesis pathway to be disrupted in this strain using the CRISPR-Cas9 system it was first transformed with an *Agrobacterium*-mediated T-DNA from plasmid pMAI23 that expresses a modified form of the *Streptomyces pyogenes Cas9* gene [21], using selection on 100 μg/ml G418. Three guide RNA constructs, for targeting the Cas9 endonuclease to the *L*. *maculans sirP*, *sirG* and *sirZ* genes, were produced. These were made by cloning the oligonucleotides “SirP”, “SirG” and “SirZ” (sequences in S1 Table) into the *Xho*I site of plasmid pMAI75 by Gibson assembly (NEBuilder HiFi Assembly Cloning Kit, New England Biolabs), and then by subcloning the gRNA constructs using restriction enzymes *Nhe*I and *Spe*I into the *Xba*I site of the plasmid pPZPnat1 [24], which confers resistance to nourseothricin when the T-DNA is transformed into *L*. *maculans*.

The T-DNAs that express the CRISPR guide RNA constructs were transformed into a sirZOE-Cas9 strain, using 100 μg/ml nourseothricin as the selective agent. In this system, incorrect repair of the Cas9 endonuclease damage introduces mutations into the target regions. Transformants were screened by PCR and restriction enzyme digests to identify those with mutations at the target sites of the three genes. Transformants were then cultured through a step of isolating a colony from a single spore to ensure nuclear homogeneity, and the gene regions amplified and analyzed using Sanger sequencing of PCR amplicons to define the mutations.

### Extraction of secondary metabolites, and their separation by thin layer chromatography or high performance liquid chromatography

25 ml CV8 liquid were inoculated with approximately 10^6^ spores of *L*. *maculans* strains. After culturing for seven days at 22°C in darkness, mycelia were removed by filtration through miracloth and the culture filtrate solution (volume approximately 20 ml) was extracted once with 20 ml ethyl acetate. The extracts were dried under a stream of nitrogen gas and the residue suspended in 200 μl pure ethanol.

For thin layer chromatography (TLC), extracts were resolved on silica gel 60 F_254_ aluminium sheets (Merck) using a 1:1 ratio of chloroform to ethyl acetate, and visualized using UV light, as in [25].

For high performance liquid chromatography (HPLC), 10 μl of each sample was injected into an Agilent 1100 machine that was run with an H_2_O-acetonitrile gradient using a Synergi 4u Fusion-RP 80A column (250 × 4.60 mm; C18; Phenomenex) as described previously [4]. Molecules eluting from the column were detected by absorbance of 240 nm UV light.

Mass spectrometry was used to confirm the identity of relevant peaks on the HPLC chromatogram. Samples peaks were manually collected and analyzed on a Thermo Q Exactive Orbitrap mass spectrometer in the positive mode.

## Results

### The genes for sirodesmin biosynthesis are down-regulated during the foliar stages of blackleg disease

**Fig 2:**
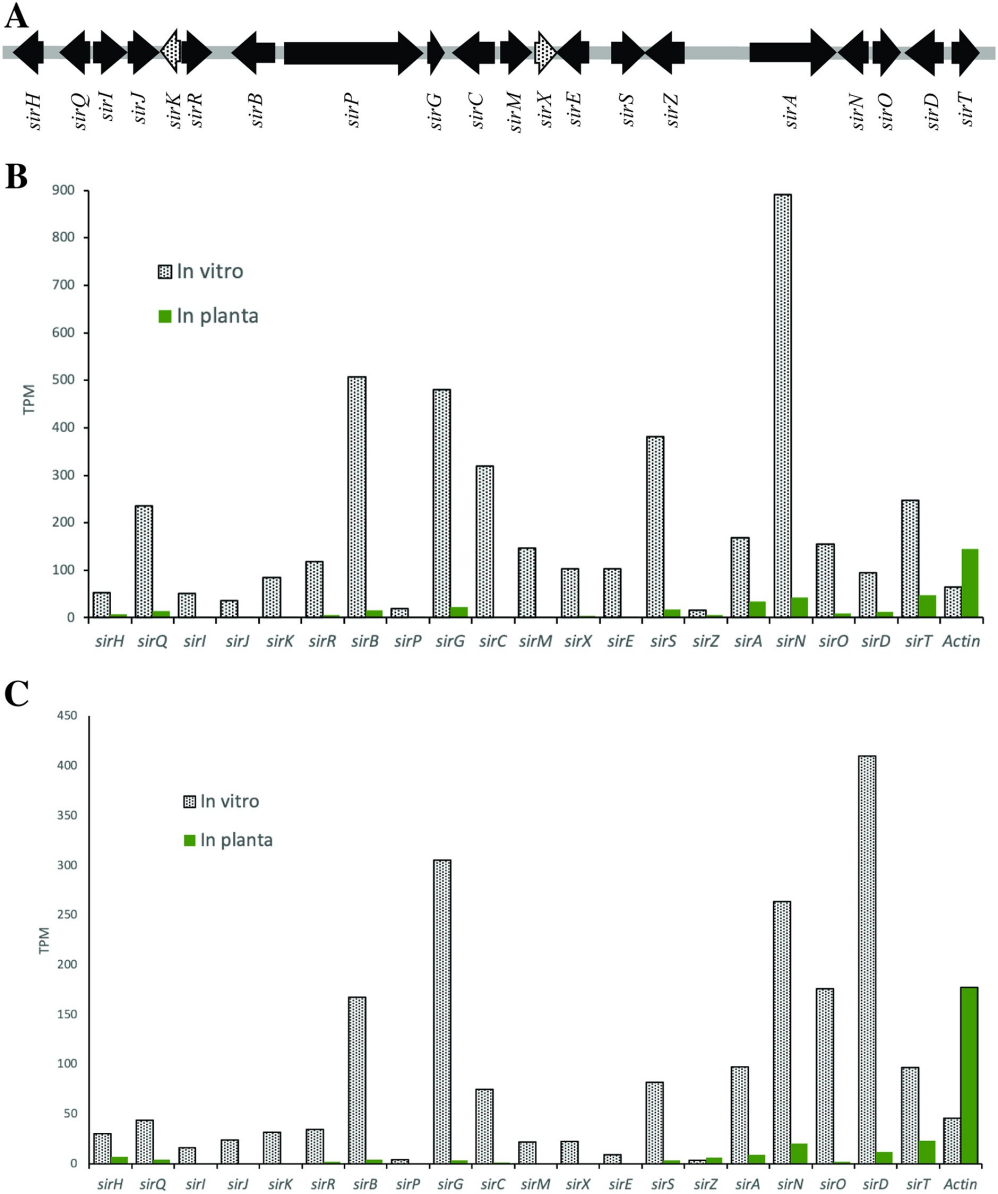
The genes for sirodesmin biosynthesis are down-regulated *in planta*. (**A**) The *sir* gene cluster consists of 20 genes, 18 of which were previously annotated (solid arrows) and two additional genes annotated as *sirK* and *sirX* (speckled arrows). (**B** and **C**) All genes in the cluster are down-regulated *in planta* compared to *in vitro*, both in the data of (**B**) Sonah *et al*. at 11 dpi and (**C**) Lowe *et al*. at 14 dpi in comparison to the actin house-keeping gene that is expressed highly *in planta* and *in vitro*.

If a protein is important for a function, its gene should be expressed when needed: two previously published RNA-seq data sets [14, 15] were re-examined for the expression of the *sir* gene cluster. All 18 annotated genes in the cluster are down-regulated during pathogenesis on cotyledons relative to *in vitro* at all time points. For example, the *in planta* data of Sonah *et al*. at 11 dpi or Lowe *et al*. at 14 dpi on cotyledons, conducted in two different countries, show the same pattern of low gene expression of the *sir* genes (Fig 2).

Mapping the transcriptomic data back to the genome sequence of *L*. *maculans* revealed two additional genes within the *sir* cluster that were not previously identified, which we name *sirK* and *sirX*. The predicted protein sequence for SirK shows homology to GliK encoded in the *Aspergillus fumigatus* gene cluster and *gliK* is essential for the synthesis of gliotoxin [26]. SirX does not show clear homology to any proteins in GenBank based on BLAST searches. Both *sirK* and *sirX* are also down-regulated during the early stages of disease, consistent with the trend of the other *sir* genes. The sequences of the two genes have been deposited in GenBank as accessions MK609857 and MK609858.

### The *L*. *maculans* actin promoter can be used to drive protein expression *in planta*

If the *sir* genes are down regulated during infection, the question was what would happen if they were expressed constitutively in strains? A candidate promoter region is actin, used in numerous gene expression studies as a standard for constitutive and strong gene expression.

**Fig 3:**
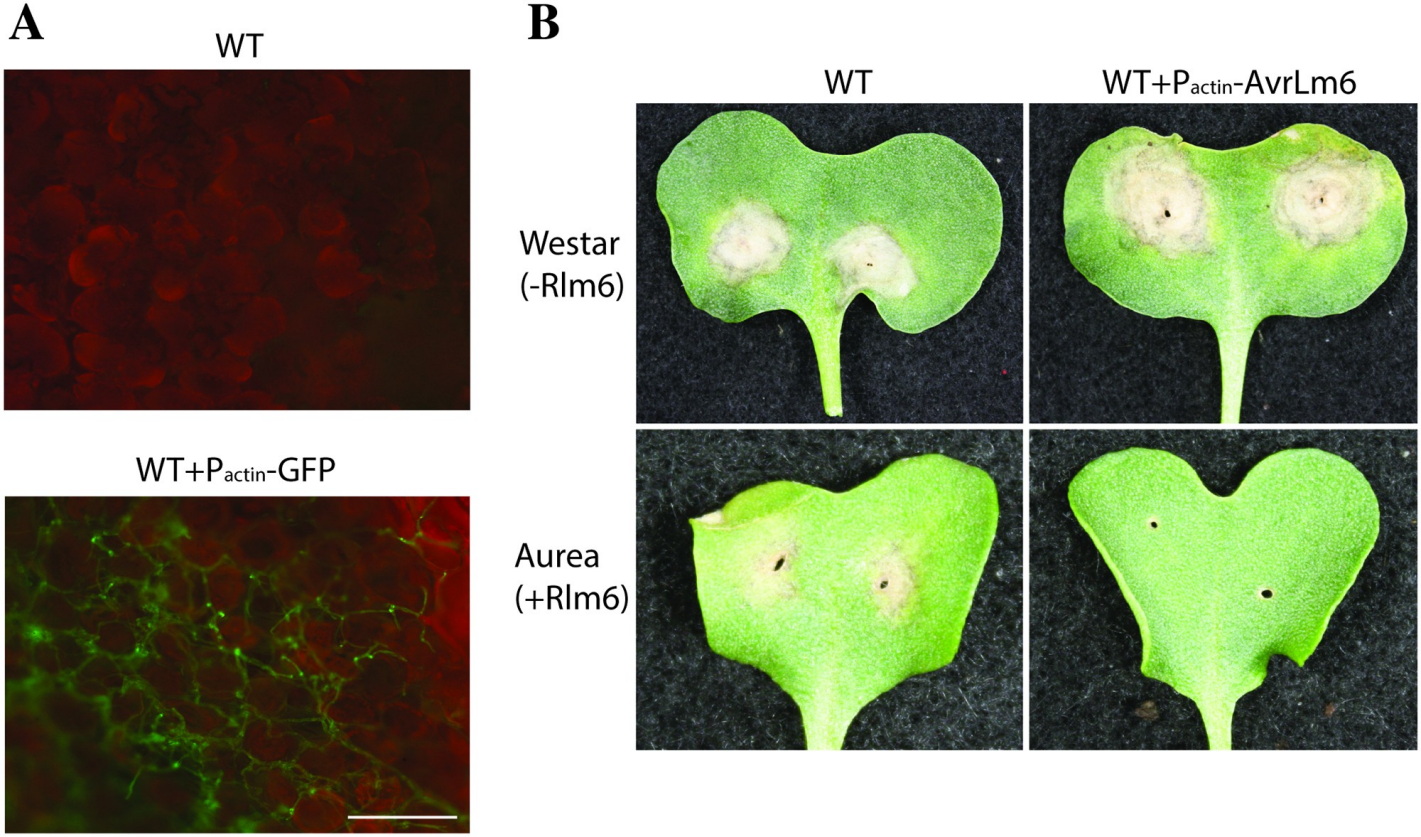
The *L*. *maculans* actin promoter can drive protein expression *in planta*. (**A**) Expression of GFP during colonization of plants 14 days post infection. Scale bar: 200 microns. (**B**) A strain expressing the avirulence protein AvrLm6 was unable to cause disease of *Brassica juncea* cv. Aurea (+Rlm6), while retaining pathogenicity on *Brassica napus* cv. Westar (no resistance genes).

To assess the ability of the *L*. *maculans* actin promoter to drive expression of proteins during early pathogenesis on cotyledons, two proteins were expressed; green fluorescent protein (GFP) and the avirulence factor AvrLm6. GFP was clearly visible as green fluorescence *in vitro* (not shown) and *in planta* (Fig 3A). Expression of AvrLm6 resulted in a loss of pathogenicity on *Brassica juncea* cv. Aurea, which contains the Rlm6 resistance gene that recognizes AvrLm6 (Fig 3B), while the wild type, which has a mutation in the *AvrLm6* avirulence gene, causes lesions. The avirulence genes are amongst those most highly expressed during the early stages of infection relative to *in vitro*, and hence these results indicate that using the actin promoter has the ability to drive expression to the levels able to impact plant disease.

### Expression of transcription factor SirZ during the early biotrophic disease stage results in a loss of pathogenicity

**Fig 4:**
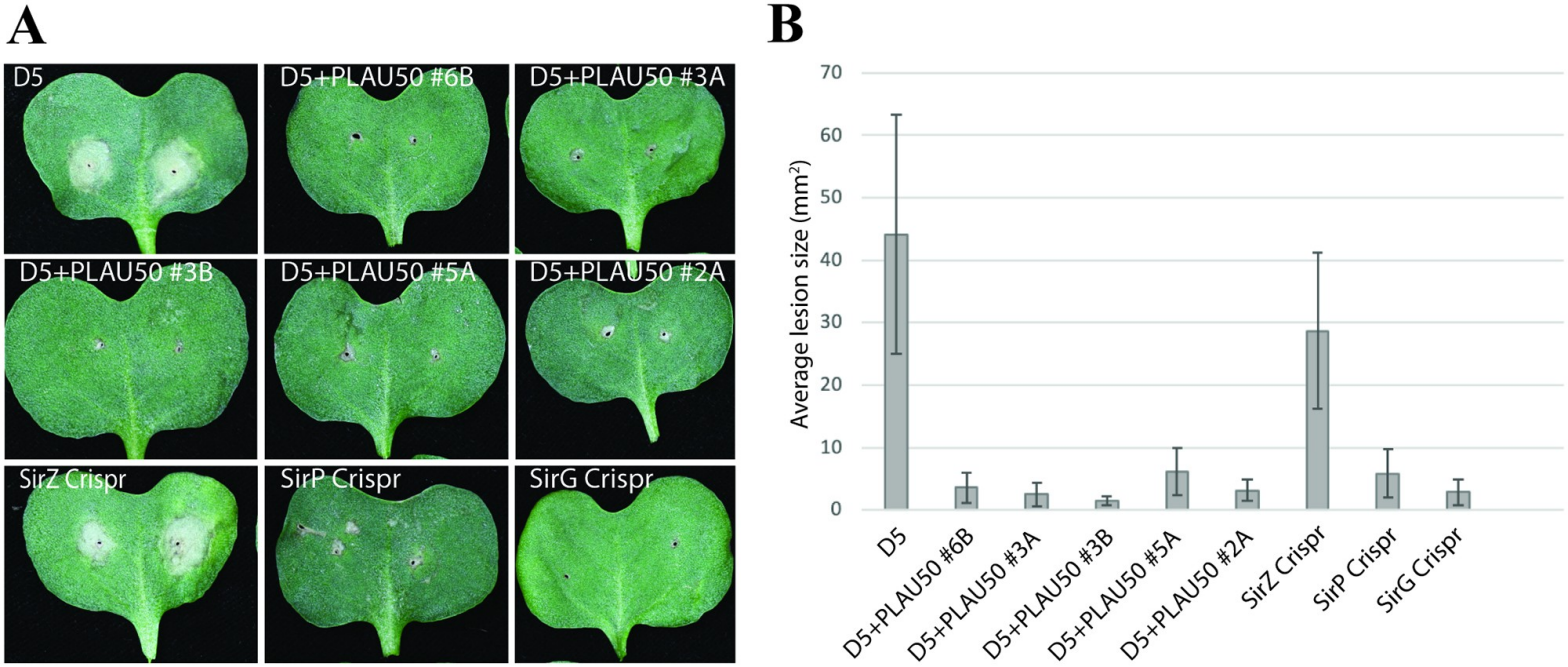
Overexpression of the SirZ transcription factor reduces pathogenicity of *L*. *maculans*. Strains were inoculated on *B*. *napus* cv. Westar and lesions analyzed 14 days later. Introduction of a construct expressing SirZ under the control of the actin promoter reduced pathogenicity of all five independent transformants tested. Disruption in one of these transformants of *sirZ*, but not *sirP* or *sirG*, restored pathogenicity. (**A**) Photographs of representative lesions on a single cotyledon inoculated with each strain. (**B**) Quantitative comparisons of lesion areas. Bars represent the average lesion size of >14 replicate inoculation points. Error bars represent +/- one standard deviation.

**Fig 5:**
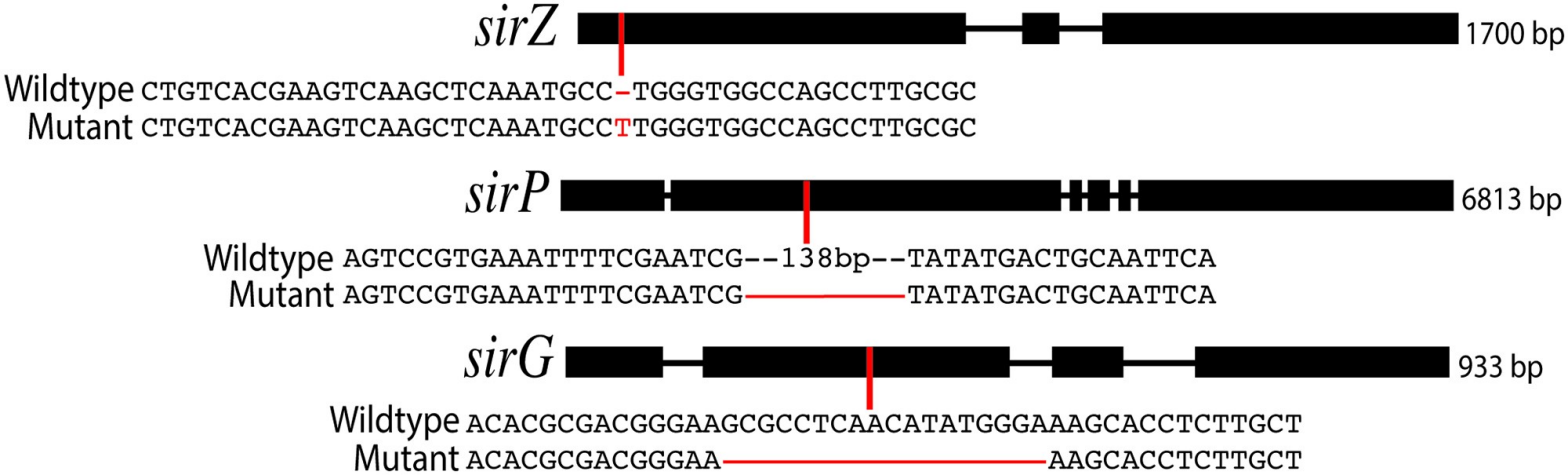
CRISPR-Cas9 gene editing of three genes in the sirodesmin gene cluster. Schematic of three genes of the *sir* cluster showing exons drawn as boxes and introns as black lines. Positions within the three genes that were targeted for CRISPR-Cas9 gene editing are marked with a red line, and DNA sequence alignments of the resultant mutant alleles with the wild type allele are shown below the corresponding gene model.

All five independent transformants expressing the actin promoter-*sirZ* construct that were tested showed a loss of pathogenicity phenotype (Fig 4). To rule out a possible inadvertent gene silencing effect of the construct, secondary metabolites were extracted from culture filtrates of the untransformed wild type and one over-expression strain (D5+sirZOE#6), resolved by thin layer chromatograph, and visualized under ultraviolet light. The over-expression strain still produced sirodesmin (data not shown). Next, to establish if the loss of pathogenicity is due to the synthesis of sirodesmin or some other effect, three genes required for sirodesmin synthesis were targeted for mutation in one representative of the over-expression strain background (i.e. strain D5+sirZOE#6). CRISPR-Cas9 constructs were transformed into this strain, and candidate mutations identified by PCR analysis. Sanger sequencing of the gene regions in these *sirZ*, *sirP* and *sirG* mutants made in the SirZ overexpressing background identified frameshift mutations indicative of loss-of-function alleles (Fig 5).

Disruption of the introduced *sirZ* allele via CRISPR-Cas9 mutation resulted in a restoration of pathogenicity (Fig 4). On the other hand, disruption of either the *sirP* or *sirG* genes, that encode enzymes for the synthesis of sirodesmin (Fig 1), did not restore pathogenicity.

Sirodesmin has other activities, such as an inhibitor of bacterial and fungal growth. We therefore tested the strains for their ability to inhibit growth of bacterium *Bacillus subtilis* or close relative of *L*. *maculans*, *Leptosphaeria biglobosa*. All strains tested maintained some level of inhibition of bacterial or fungal growth, and which was variable between different experiments (data not shown).

### Over-expression of SirZ results in a decreased growth rate that does not account for the loss in pathogenicity

**Fig 6:**
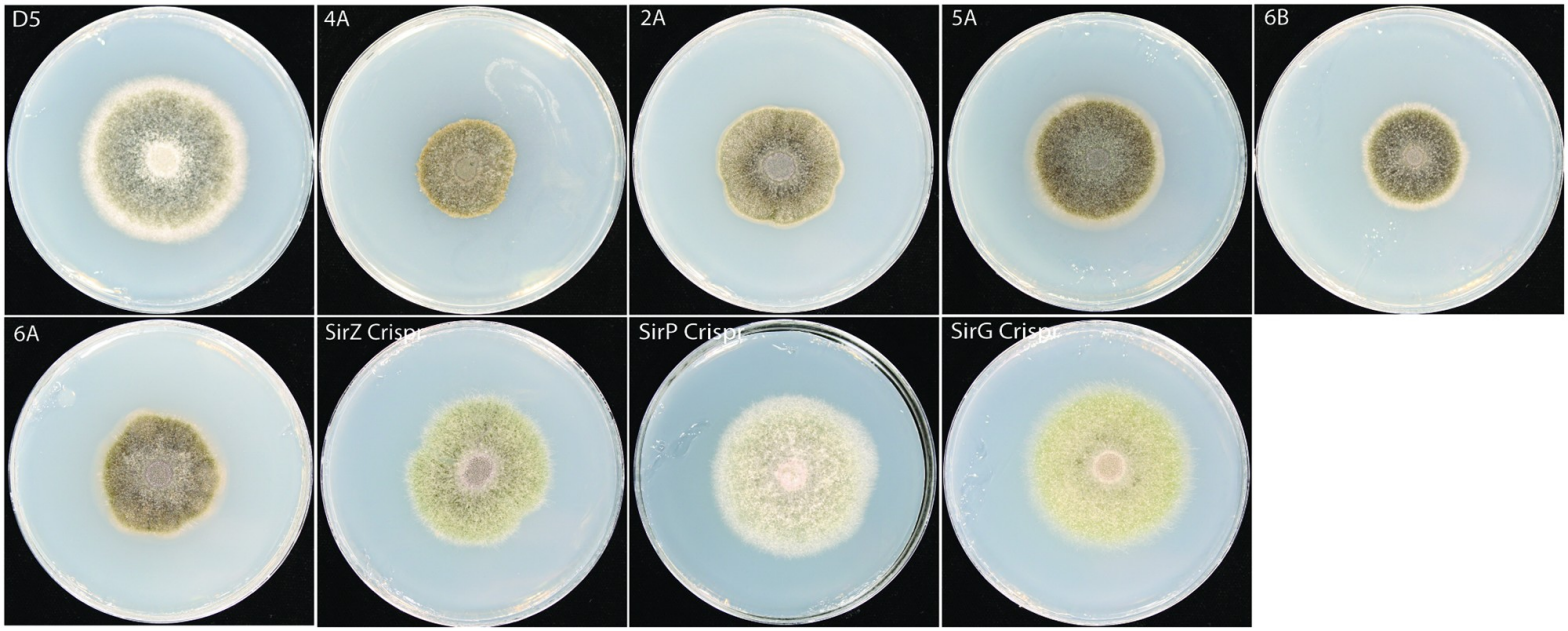
*In vitro* growth is reduced in the SirZ overexpression strains. Disruption of *sirZ*, *sirP* or *sirG* restored the growth rate. Strains were grown for 10 days on CV8 media. Plates are 90 mm in diameter.

The five actin promoter-*sirZ* transformants showed a slight reduction in growth rate and darkening of colony color (Fig 6). Disruption of *sirZ* as well as *sirG* and *sirP* reversed this effect (Fig 6), suggesting that this phenotype is attributed to the production of sirodesmin. Because the growth defect is reverted in the *sirG* or *sirP* mutants, yet these strains remain non-pathogenic, this indicates that the loss of pathogenicity caused by *sirZ* overexpression is not simply due to slower growth.

### HPLC analysis of secondary metabolites in wild type and mutant strains confirms gene functions

**Fig 7:**
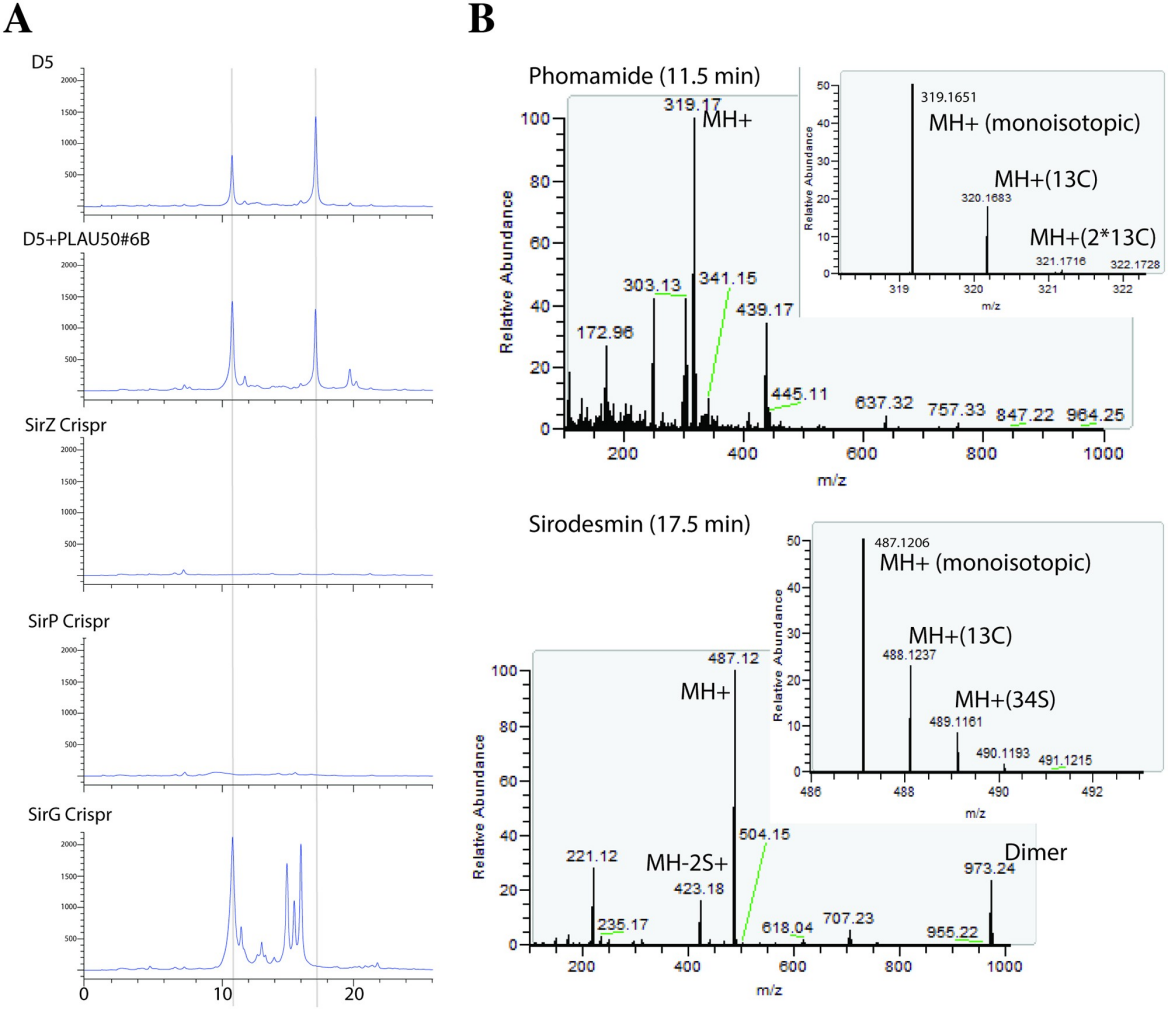
SirG and SirP are required for sirodesmin synthesis. (**A**) HPLC analysis of culture filtrates from the wild type and *sirZ* overexpression (D5+PLAU50#6) strains revealed two major peaks at approximately 11.5 and 17.5 mins, whose presence changes when *sirP* or *sirG* are mutated. (**B**) Fourier transform mass spectra were generated from 1 ml fractions collected at 11.5 min and 17.5 mins from the wild type strain. The presence of *m/z* 487.12 (M+H) and a fragment ion characteristic of sirodesmin *m/z* 423.18 (M+H-2S) were found in the 17.5 minute fraction. The presence of *m/z* 319.17 (M+H) from the 11.5 min fraction corresponds to phomamide. The *sirZ* and *sirP* mutants produced neither molecule. The *sirG* mutant produced phomamide, in addition to a number of novel metabolites.

HPLC analysis revealed that the *sirZ* and *sirP* mutants produced neither sirodesmin nor its precursor phomamide, thus confirming the function of these genes had been abolished (Fig 7A). The *sirG* mutant produced phomamide but not sirodesmin, and the *sirG* mutant produced a number of additional products. This is consistent with the position of where the enzymes SirP and SirG act in the pathway for sirodesmin synthesis (Fig 1). Mass-spectra obtained from selected HPLC fractions confirmed that the 11.5 min peak represents phomamide and the 17.5 min peak represents sirodesmin PL (Fig 7B).

## Discussion

The transcription factor SirZ, which regulates the synthesis of the major secondary metabolite produced by *L*. *maculans* [8, 9], was constitutively expressed and this blocked pathogenicity in cotyledon infection assays (Fig 4). Because the strain is no longer pathogenic, the transcript levels of *sirZ* itself during infections could not be measured due to the limited amount of fungal biomass. However, as a control the same promoter used to drive expression of GFP or the avirulence factor AvrLm6 both led to the functional expression of high levels of these proteins. Disruption of the SirZ overexpression allele, via CRISPR/Cas9, restored pathogenicity, indicating that the loss of pathogenicity was related to the expression of SirZ.

*In vitro* the SirZ constitutive expression strains had slower radial growth rates compared to the wild type, which may reflect autotoxicity (Fig 6). However, this growth rate is not the cause of the reduction in pathogenicity. First, *L*. *maculans* wild isolates exhibit a range of growth rates yet cause equal lesion sizes on cotyledons (A.P. Van de Wouw, pers. commun). Second, we have isolated mutants with vegetative growth defects yet remain fully pathogenic [19]. Third, disruption of either *sirP* or *sirG* resulted in restoration back to wild type growth rate but not to wild type pathogenicity. Hence, the overexpression of *sirZ* leads to a true pathogenicity defect rather than a general reduction in strain fitness.

To explore the mechanism behind the loss of pathogenicity when *sirZ* is overexpressed, we selected two genes in the sirodesmin biosynthesis cluster for disruption using CRISPR/Cas9: *sirP* and *sirG*. The *sirP* gene was chosen because it has previously been shown to be required for sirodesmin production and is expected to act early in the biosynthetic pathway, which agrees with the fact we did not detect sirodesmin or phomamide production in the *sirP* mutant. The *sirG* gene was chosen because this gene has not previously been demonstrated to be essential for sirodesmin production, although homology to *gliG* in the gliotoxin biosynthetic pathway of *A*. *fumigatus* suggested that it was responsible for adding the sulfur atoms to the molecule by acting downstream of phomamide [6]. The phytotoxicity of sirodesmin (as well as other epipolythiodioxopiperazines) has been attributed to the disulfide bridge that enables these toxins to cross-link proteins via cysteine residues [27]. The HPLC chromatograph of the *sirG* disrupted strain supports such a role as phomamide, but not sirodesmin, was present in the culture filtrates. In addition to phomamide, a number of novel peaks were observed in the *sirG* mutant. This is similar to the *gliG* deletion strain of *A*. *fumigatus* that produced compounds not observed in either the wild type or *gliP* (homologous to *L*. *maculans sirP*) deletion strain [6].

Given that the strains in which *sirG* and *sirP* were mutated in the *sirZ* overexpression background remained non-pathogenic we conclude that the loss of pathogenicity due to SirZ overexpression is not due to the over-production of sirodesmin. Two possible explanations for this unexpected finding are that expression of one of the sirodesmin biosynthesis pathway genes is causing the loss of pathogenicity or that SirZ regulates additional targets outside of the cluster. The possibility of the second explanation is supported by the fact that regulatory cross talk between biosynthetic gene clusters has been reported previously in other fungi. A number of variations have been observed. These can be situations in which a transcription factor can regulate two gene clusters, as for ScpR and the RsmA-AflR regulon in *Aspergillus nidulans* [28, 29] or FapR in *A*. *fumigatus* [30]. Some transcription factors within a cluster can have widespread influence on secondary metabolism, such as Tri6 in *Trichoderma arundinaceum* that regulates numerous secondary metabolite clusters although this may also be via alteration of the mevalonate biosynthetic genes [30]. The consequences of overexpression can thus be complex, e.g. if a transcriptional regulator also influences the availability of the substrates for secondary metabolites. Or, another curious situation is the transcription factor HasA that regulates hexadehydroastechrome synthesis yet also controls genes involved in iron homeostasis. The consequences of overexpression of HasA, most likely mediated via changes in metal homeostasis, then impacts the expression of 14 gene clusters in addition to the *has* cluster [31]. One final example worth noting is the case of the synthesis of azophilones, which requires the activities of two separate gene clusters in *Aspergillus terreus*. In this case, a transcription factor in one of the clusters acts to induce the expression of two other transcription factors one each found in the two clusters [32]. In light of these examples, in either the case of another *sir* gene or secondary metabolite being involved, these findings from *L*. *maculans* are another caution against assuming that phenotypes due to overexpression of putative pathway specific transcription factors can be solely attributed to increased metabolite production.

Discovering which genes, other than those in the sirodesmin gene cluster, are regulated when *sirZ* is over-expressed should reveal new genes required for disease development. A bioinformatic analysis of the genes in the epipolythiodioxopiperazine synthesis gene clusters from three species revealed a consensus motif of TCGGNNNCCGA is in the promoters of eight of the *L*. *maculans sir* genes [8], and it is hypothesized that SirZ binds to this motif. We therefore examined the secondary metabolite core enzymes and surrounding associated genes in the *L*. *maculans* genome, as defined in the MycoCosm database [33], by searching manually for the relaxed motif of CGGNNNCCG. The genome is predicted to encode 13 non-ribosomal peptide synthases, 12 polyketide synthases, and two terpene synthases [20]. Eight motifs were found in the *sir* cluster, as previously reported [8]. A single candidate motif was associated with six other gene clusters. Four weaker candidates were in the gene promoter of the putative major facilitator superfamily transporter for the ‘NPS3 cluster’, between a fatty acid transporter and hypothetical protein in the NPS11 cluster, the monooxygenase in the NPS2 cluster, and between the major facilitator superfamily transporter and NPS1. The two most conserved motifs were for the monooxygenase in the PKS1 cluster and between a hypothetical protein and NPS4. None of these gene clusters have defined functions. Hence, the identification of other SirZ-regulated genes may require RNA-sequencing comparisons or could also be addressed by a mutant screen seeking a restoration to wild type pathogenicity.

Sirodesmin is the best characterized secondary metabolite of *L*. *maculans*, and yet this study further complicates its possible functions in the biology of this and other fungi. The metabolite is produced during *in vitro* growth and in stem lesions, yet *sir* gene expression is turned down during the early stages of plant infection. These findings tend to support a hypothesis that the fungus produces the metabolite for competition with other microbes as part of its saprotrophic growth. Sirodesmin has other activities, such as inhibition of bacteria or fungi [12, 34]. The strains created here all still maintained the ability to inhibit bacterial or fungal growth. However, these types of studies on living cultures, rather than using purified metabolites, can be problematic in cases whereby bacterial-fungal or fungal-fungal interactions trigger the production of secondary metabolites potentially in both organisms [35]. Similarly to *L*. *maculans*, the ‘primary’ functions of other ETPs have been difficult to assess. For instance, gliotoxin was isolated as an antifungal agent, is immunosuppressive, has anti-tumor properties and in specific conditions contributes to the virulence of *A*. *fumigatus* (the inconsistent findings are reviewed in [36]). Roles for gliotoxin are well illustrated in *Trichoderma virens*, where the *gliP* mutant loses its mycoparasitic activity against the oomycete *Pythium ultimum* and the fungus *Sclerotinia sclerotiorum*, and is less pathogenic against the insect *Galleria mellonella* [37].

This study suggests a promising new direction for future studies on *L*. *maculans* on the investigation of genes that are not normally expressed during disease. Most previous work on *L*. *maculans* has focused on either avirulence genes or on pathogenicity genes, which are two groups of genes that are normally expressed during disease. However, RNA-sequencing data [14, 15] show that a large number of genes are expressed at a high level during *in vitro* growth but at a very low level during the early biotrophic stages of disease. Despite having been probed for a role in pathogenicity [4, 8, 12, 24] the genes of the sirodesmin biosynthetic cluster fall into this group. It is possible that many such genes are not only dispensable at this stage of disease but moreover that their expression during this stage would be deleterious. We have demonstrated this to be the case for *sirZ*. If strategies can be developed to cause pathogenic fungi to miss-express such genes this would be a powerful tool in the fight against plant disease.

## Supporting information

S1 Table Oligonucleotide primers used in this study. (PDF)
