# Supplementary material for: Constitutive expression of transcription factor SirZ blocks pathogenicity in Leptosphaeria maculans independently of sirodesmin production
Source: PLoS One. 2021 Jun 10;16(6):e0252333. doi: 10.1371/journal.pone.0252333 (PMC8191991; doi:10.1371/journal.pone.0252333)
Supplement: S1 Table — (PDF) [file pone.0252333.s001.pdf]

S1 Table. Oligonucleotide primers used in this study.

Name	Sequence (5´-3´)
AU226	TCGAAACCTAATCAATCAACATGTCGCCAGCACCGCCGC
AU227	TGCTCATAGTCACATCCCTCAGCAGCCCACCTGTTTTTCC
AU24	CGAAACCTAATCAATCAACATGGTGATTTACCTACCCC
AU27	GCTCATAGTCACATCCCTCATTGGATTTGTCCTTCCC
AU28	CGAAACCTAATCAATCAACATGGTGAGCAAGGGCGAGG
AU31	GCTCATAGTCACATCCCTCACTTGTACAGCTCGTCCATG
SirZ	GAAACCTAATCAATCAACCTTGACCTGATGAGTCCGTGAGGACGAAACGAGTAAGCTC GTCGTCAAGCTCAAATGCCTGGGGTTTTAGAGCTAGAAATAGC
SirG	GAAACCTAATCAATCAACTTCCCGCTGATGAGTCCGTGAGGACGAAACGAGTAAGCTC GTCCGGGAAGCGCCTCAACATATGTTTTAGAGCTAGAAATAGC
SirP	GAAACCTAATCAATCAACGAGTCGCTGATGAGTCCGTGAGGACGAAACGAGTAAGCTC GTCCGACTCGGAGGAAGCAGATCGTTTTAGAGCTAGAAATAGC
